# Effect of high-copper diet on transference of *bla*_CTX−M_ genes among *Escherichia coli* strains in rats' intestine

**DOI:** 10.3389/fvets.2023.1127816

**Published:** 2023-03-09

**Authors:** Kang Liu, Linqian Li, Mengwei Weng, Feng Zhang, Rong Guo, Jinhu Huang, Wen Yao

**Affiliations:** ^1^College of Animal Science and Technology, Nanjing Agricultural University, Nanjing, Jiangsu, China; ^2^College of Veterinary Medicine, Nanjing Agricultural University, Nanjing, Jiangsu, China; ^3^Key Lab of Animal Physiology and Biochemistry, Ministry of Agriculture and Rural Affairs of the People's Republic of China, Nanjing Agricultural University, Nanjing, Jiangsu, China

**Keywords:** copper, ceftiofur, *E. coil*, conjugation transfer, *bla*_CTX−M−1_

## Abstract

Both ceftiofur (CTO) and high copper are widely utilized in animal production in China, and the occurrence of CTX-M-carrying *Escherichia coli* in food-producing animals is increasing. There are some specific associations between in-feed high-level copper and antibiotic resistance, but research in Gram-negative bacteria such as *E. coli* remains scarce. This study aimed to evaluate the effect of high-copper diet on the horizontal transfer of *bla*_CTX−M−1_ among *E. coli*. A total of 32 male SPF rats aged 21 days were randomly assigned to the following four groups: control (6 mg/kg in-feed copper, C^−^), high copper (240 mg/kg in-feed copper, H^−^), CTO (6 mg/kg in-feed copper with oral CTO administration, C^+^), and high copper plus CTO (240 mg/kg in-feed copper with oral CTO administration, H^+^). All rats were orally inoculated with an *E. coli* strain harboring a conjugative plasmid carrying *bla*_CTX−M−1_, and the C^+^ and H^+^ groups were given 10 mg/kg of body weight (BW) CTO hydrochloride at 26, 27, and 28 days, while the C^−^ and H^−^ groups were given salad oil at the same dose. Fecal samples collected at different time points were used for the enumeration of *E. coli* on Mac plates or for molecular analysis using PCR, pulsed-field gel electrophoresis (PFGE), S1-PFGE, and Southern-blot hybridization. The results showed that the number of the *bla*_CTX−M−1_ gene in the H^−^ group was higher and that the loss speed of this gene was slower compared with the C^−^ group. After administration of CTO, the counts of cefotaxime-resistant *E. coli* were significantly higher in the C^+^ group than that in the corresponding control group (C^+^ vs. C^−^; H^+^ vs. H^−^). In the *in vitro* test, the results showed that the transfer rates of the conjugation induced by the H^−^ (12 mmol/L) group were significantly higher than that of low copper (2 mmol/L) group. The indigenous sensitive isolates, which were homologous to the *bla*_CTX−M_-positive isolates of rat feces, were found by PFGE. The further analysis of S1-PFGE and Southern-blot hybridization confirmed that the *bla*_CTX−M−1_ gene in new transconjugants was derived from the inoculated strain. Taken together, high-copper diet facilitates the horizontal transfer and maintenance of the resistant genes in the intestine of rats, although the effects of antibiotics on bacterial resistance appearance and maintenance are more obvious.

## Introduction

Ceftiofur (CTO), an extended-spectrum third-generation cephalosporin, was authorized for the prevention and treatment of animals' bacterial diseases in China, which accelerated the occurrence and dissemination of extended-spectrum-βlactamases (ESBLs) producing *Escherichia coli* (*E. coli*) in food-producing animals (1, 2). ESBLs can disseminate not only through the clonal expansion of resistant bacteria, in which a resistant gene is passed vertically into the offspring but also through horizontal transfer (through conjugation, transformation, or transduction) of the resistance genes that are located on mobile genetic elements (plasmids, transposons, and integrons) (3), which pose a particularly serious public health threat worldwide (4, 5). The most frequently reported ESBLs are CTX-M-type enzymes, which can be divided into five major groups (CTX-M-1, CTX-M-2, CTX-M-8, CTX-M-9, and CTX-M-25) (6).

Copper is one of the essential trace elements, and in-feed high-level copper has been reported to promote growth, increase feed intake, and improve feed utilization (7) and is widely used for feed additives in livestock production for decades. Because of the low absorption of copper, the intestinal level of copper is much higher than that in the feed level, with an accumulation of more than sixfold (8), which indicates that bacteria in the intestinal environment are always facing the pressure of high copper. Resistance to copper has been reported around the world (9–11).

Horizontal gene transfer is considered an important mechanism for the widespread distribution of antibiotic resistance genes in bacteria (12). Some mobile genetic elements, such as coupled plasmids, are highly transferable, and when antibiotic resistance genes are located on the same genetic element together with metal resistance coding genes, heavy metal resistance and antibiotic resistance coding genes are easily transferred together to other bacteria (13). Some studies have found that, in Gram-positive enterococci, copper may affect the range of resistance to special antibiotics (14, 15), and the co-transfer of genes encoding Cu resistance and genes encoding numerous antibiotic resistances has been corroborated (16, 17). However, the specific association between in-feed high-level copper and antibiotic resistance in Gram-negative bacteria such as *E. coli* remains scarcely researched. Does the high copper content in feed act as a selective pressure and play an important role in the maintenance and transfer of the CTX-M-type ESBL gene in *E. coli*? This study aimed to use rats as an animal model in order to evaluate the impact of long-term in-feed high-level copper on horizontal transfer of *bla*_CTX−M−1_ *in vivo* by orally giving a conjugative *E. coli* strain with a *bla*_CTX−M−1_-positive plasmid.

## Methods

### Preparation and characterization of the inoculum strain

A CTO-resistant *E. coli* isolate harboring *bla*_CTX−M−9_ and *bla*_CTX−M−1_ acting as a donor strain was isolated and identified from Sprague-Dawley (SD) rats which fed on a high-copper diet (240 mg·kg^−1^) for 8 weeks and then orally accepted CTO hydrochloride one time a day for 6 days in our previous study. EC600, an *E. coli* strain that showed white colonies on MacConkey (MC) agar, was susceptible to cephalosporins and was used as the recipient strain. It was kindly supplied by Hong Kong Polytechnic University Shenzhen Research Institute.

The conjugation used the filtermating method as described in a previous study (18). Overnight cultures of the donor strain and recipient strain were mixed in a ratio of 1:4 in LB media and then transferred to a filter membrane on an LB plate and incubated for 16 h at 37°C. The flushed fluid of the filter membrane was then inoculated on the MC plates containing cefotaxime sodium (Dalian Mei Lun Biotechnology Co., Ltd., China, 2 μg/ml) for 24 h at 37°C. The white colonies on MC plates were tested for antimicrobial susceptibility and the presence of *bla*_CTX−M_ genes.

Plasmid DNA from a successful transconjugant, EC600 (TC4-2), was extracted using TruSeq™ DNA Sample Prep Kit (Illumina, USA) and sequenced with the Illumina NextSeq 500 platform and the PacBio RSII single-molecule real-time sequencing platform and then annotated with the RAST tool and the NCBI Prokaryotic Genome Annotation Pipeline. This successful transconjugant was used as an inoculum in this study.

### Conjugation assays *in vitro*

In the *in vitro* test, the inoculation strain was used as a donor and the CTO-susceptible *E. coli* isolated from rats was used as a recipient. The horizontal-transfer efficiencies were assessed by performing conjugation using the filtermating method as described in a previous study. The experimental groups were classified as follows: the non-treated control group (LB media containing 2 mM copper) and the high copper-treated group (LB media containing 12 mM copper).

### Animals and experimental design

The animal experiment was conducted at the Experimental Animal Center of Nanjing Agricultural University, Jiangsu Province, China. A total of 32 specific pathogen-free (SPF) weaning male rats (21 days old) were kept in a barrier environment for an 8-week feeding trial. Strict biosecurity measures were implemented to avoid contamination of the rats. All the rats were randomly assigned to two control groups (C^−^/C^+^) and two treatment groups (H^−^/H^+^) and then underwent a 3-day plate detection procedure to guarantee the absence of the existence of CTO-resistant *E. coli*. Rats in the C^−^/C^+^ groups were fed a 6 mg/kg copper diet, and rats in the H^−^/H^+^ groups were fed a 240 mg/kg copper diet. All rats were orally given 1 ml of transconjugant inoculum *E. coli* TC4-2 (10^8^ CFU/ml) on D20, D22, and D24 during the feeding trial. Rats in the C^+^/H^+^ groups were orally given 1 ml of CTO hydrochloride (10 mg/kg of BW) one time a day for 3 days from D26 to D28, while the same volume of edible oil was given to rats in C^−^/H^−^ groups at the same time. Individual fecal samples were collected from all rats on D1, D2, D3, D14, D16, D18, D21, D23, D25, D27, D28, D31, D33, D35, D39, D43, and D50. Rats' feces were used for Mac *E. coli* enumeration, isolation, and identification of sensitive or resistant *E. coli* and were stored at −20°C for other analyses.

Diet was created with reference to test Rat Feed Formula and Experimental Rat Synthetic Feed Formula (AIN-93G) of the American Chemical Analytical Chemists Association (AOAC). The copper level in the feed and fecal samples was measured using an Optima 2100DV Inductively Coupled Plasma Atomic Emission Spectrometer (PerkinElmer, USA) (19).

### Antibiotic susceptibility testing of bacteria in rats' feces

To enumerate the sensitive and resistant *E. coli*, 0.1 g of fecal sample was suspended, serially diluted tenfold in sterile normal saline, and plated on Mac agar plates containing zero (Mac plate) or 2 μg/ml cefotaxime sodium (cef-Mac plate). The plates were incubated at 37°C for 24 h. The total colony counts on Mac agar and the red colony counts on the cef-Mac plate were recorded to determine the total *E. coli* number and the transconjugant *E. coli* number of colony forming units per gram of fecal sample (CFU/g). The ratio of resistant CFUs was measured as the number of CFUs on plates containing 2 μg/ml cefotaxime sodium relative to the number of CFUs on plates without antibiotics.

Four single colonies with typical *E. coli* morphology per Mac agar plate (per collective sample on D1, D2, D3, D14, D16, and D18) were picked and stored at −20°C. On other sampling days, a single colony with typical *E. coli* morphology per cef-Mac plate was picked and streaked on cef-Mac plates. The plates were incubated at 37°C for 24 h; afterward, four isolates were randomly chosen per fecal sample to store at −20°C for further analysis.

Antimicrobial susceptibility was determined by the minimum inhibitory concentrations (MICs) of different antimicrobials that were performed by the Mueller-Hinton (MH) broth microdilution method according to Clinical and Laboratory Standards Institute (CLSI) recommendations using *E. coli* ATCC 25,922 as quality control (20). Standard antibiotics were obtained from Sangon Biotech Company (Shanghai, China), except CTO (Dalian Meilun Bio-Tech Co., Ltd., Dalian, China). The MICs of *E. coli* for copper were also determined according to CLSI, as described previously (21, 22). CuSO_4_ concentrations used were 0, 2, 4, 8, 12, 16, 20, 24, 28, and 32 mM. The addition of CuSO_4_ to the medium caused a significant drop in pH (23); therefore, the medium was adjusted to a pH of 7.0 by adding NaOH prior to bacterial inoculation.

### PCR detection of *bla*_CTX-M_ genes and copper resistance genes

The detection of *bla*_CTX−M_ genes and copper resistance genes in *E. coli* isolated from CTX-Mac plates was also conducted. The resistance genes in the *E. coli* isolates were determined by PCR, as previously described (24). The primers of *pco*A and *pco*D genes were obtained from the Hong Kong Polytechnic University, Shenzhen Research Institute, and *pco*B*, pco*C*, pco*E, and *Cue*O primer sequences refer to reference literature (25).

DNA was prepared from colonies on MC agar plates. A single colony picked from an MC plate was inoculated in a 1.5-ml centrifuge tube containing 150 μl of sterile ultrapure water, placed in a water bath at 95°C for 10 min and then in an ice bath for 10 min, and centrifuged at 12,800 rpm for 2 min, and the supernatant was used as a DNA template.

Each DNA extract was quantified using a Nanodrop 2000 spectrophotometer (Thermo Scientific, America) and was then adjusted to proper concentration. *E. coli* isolates were tested for *bla*_CTX−M_, *Cue*O, and *pco* genes from DNA extracted by heating each bacterial suspension in 150 ml of nuclease-free water at 95°C for 10 min and then placing it in an ice bath for 10 min. After centrifugation at 12,800 rpm for 2 min, the lysate was separated and used as a template for PCR. Primers and positive controls (and their sources) are shown in Table 1. The PCR mixture of 20 μl consisted of 10 μl of Premix Ex Taq, 1 μl of primer mix, 1 μl of DNA template, and 1 μl of nuclease-free water. PCR conditions for β-lactamase genes were as follows: initial activation at 94°C for 10 min, followed by 30 cycles of denaturation at 94°C for 40 s, annealing for 40 s, and extension at 72°C for 40 s, followed by a final extension at 72°C for 1 min. PCR conditions for *pco* and *Cue*O genes were as follows: initial activation at 95°C for 10 min, followed by 35 cycles of denaturation at 95°C for 1 min, annealing for 1 min, and extension at 72°C for 5 min, followed by a final extension at 72°C for 1 min.

**Table 1:** Primer sequence of PCR.

**Resistance gene**	**Primer sequence (5′-3′)**	**Tm (°C)**	**Product size (bp)**
*bla*_CTX−M−1_	F:TTAGGAARTGTGCCGCTGYA	60	688
	R:CGATATCGTTGGTGGTRCCAT		
*bla*_CTX−M−9_	F:TCAAGCCTGCCGATCTGGT	60	561
	R:TGATTCTCGCCGCTGAAG		
*pco*A	F:CGTCTCGACGAACTTTCCTG	60	1,791
	R:GGACTTCACGAAACATTCCC		
*pco*B	F: GTCGCCGGTTTGTTTACCT	60	799
	R: TTTTCGCCATATCGGATGTT		
*pco*C	F: TTCTTACAGGTGGCCTCGTT	60	334
	R: CCGGTAATAGGGTGCGTATC		
*pco*D	F:CAGGAACGGTGATTGTTGTA	56	702
	R:CCGTAAAATCAAAGGGCTTA		
*pco*E	F: GTGGGGCAGCTTTTGCTCAGTCCAGTGA	63	385
	R: CGAAGCTTTCTTGCCTGCGTCTGATGTG		
*Cue*O	F: GCATGCAACGTCGTGATTTC	55	1,551
	R: GCTTATACCGTAAACCCTAA		

### Genotyping and Southern blot for homogenous isolates

After digestion with SmaI, the pulsed-field gel electrophoresis (PFGE) profiles of a few isolates were compared to the profile of the inoculated *E. coli* TC4-2 strain to analyze the homology using the BioNumerics software (Applied Maths, Belgium), as described previously (26). The results were interpreted according to the criteria of Tenover et al. (27).

Furthermore, the transconjugants and the corresponding recipient strains, as well as the inoculation strain TC4-2 and the donor strain No. 4 (the donor strain of the inoculation strain) were subjected to S1-PFGE and Southern-blot hybridization. The specific method refers to the operating procedures of Xie et al. (28).

### Statistical analysis

The *bla*_CTX−M−1_ gene copy numbers and individual numbers of CTO-resistant *E. coli* were log10-transformed, and differences between the non-treated and treated groups were analyzed using a one-way ANOVA or the Kruskal–Wallis test. Copper levels in rat feed and fecal samples were measured using the Kruskal–Wallis test. Conjugation frequency *in vitro* was analyzed using the Wilcoxon test. Distributions were compared using the chi-square test or Fisher's exact test. For all tests, a value of *P* of < 0.05 was considered a statistically significant difference. Statistical analyses were performed using the SPSS version 22.0 software (IBM Inc., Chicago, IL, USA).

## Results

### The inoculated strain contained *bla*_CTX-M-1_, *bla*_CTX-TEM_, and *fos* A

**Figure 1:**
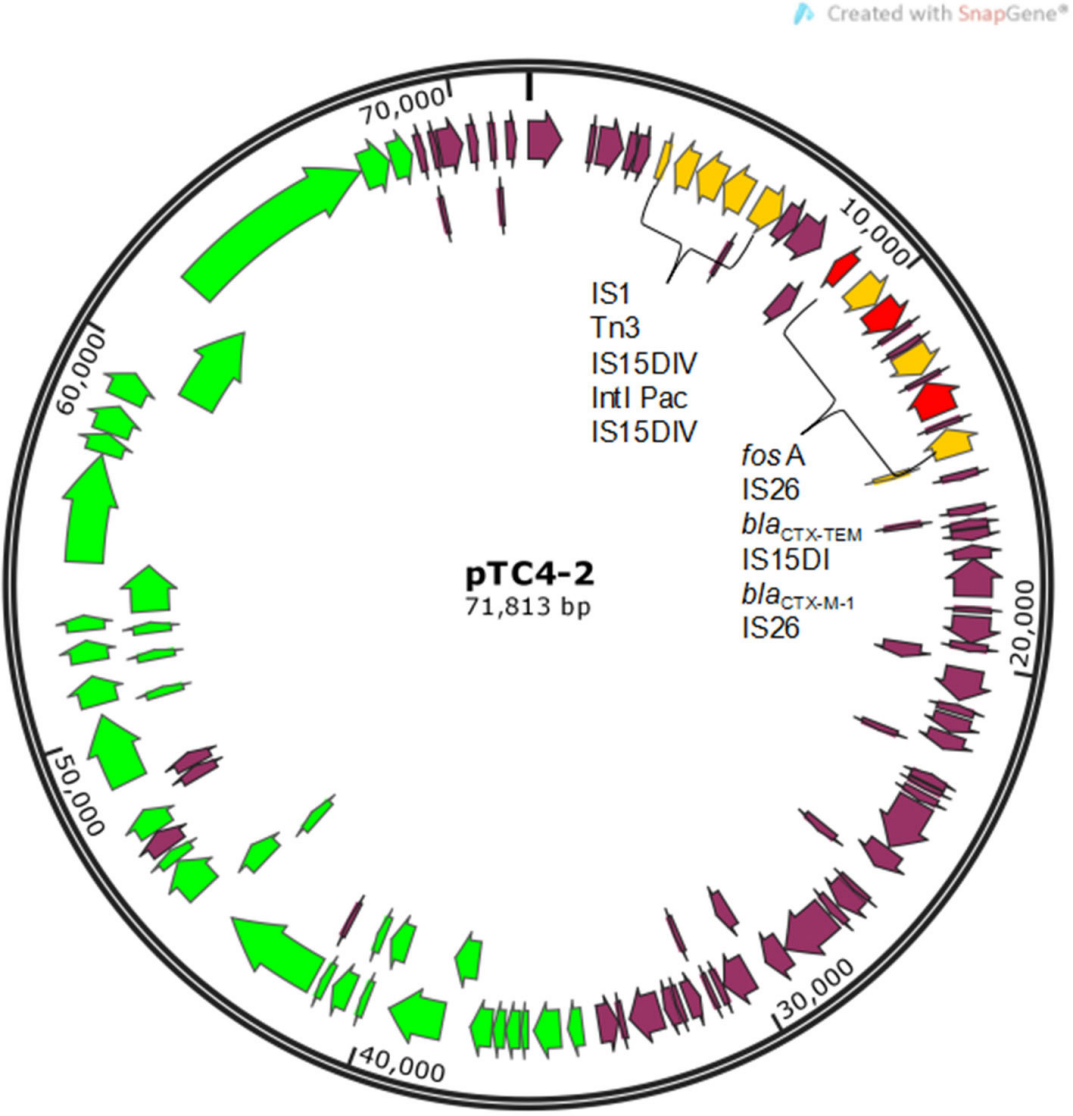
Gene map of conjugative plasmid pTC4-2. Yellow arrows represent mobile elements. Red arrows represent resistance genes. Green arrows represent genes related to conjugal transfer functions.

An engineering strain, EC600, which is resistant to rifampicin but susceptible to cephalosporins, was used as the recipient strain, and a strain carrying *bla*_CTX−M−9_ and *bla*_CTX−M−1_, which was obtained from our previous study, was used as the donor strain. One of the fourteen successful transconjugant isolates, named TC4-2, was used as inoculum in this study after MICs and PCR determination. This inoculum strain TC4-2 obtained the *bla*_CTX−M−1_ gene from the donor strain and resistant phenotypes from both the donor and recipient strains, but the *bla*_CTX−M−9_ gene was unable to transmit (Tables 2, 3). EC600, donor strain, and TC4-2 all have a copper-resistant phenotype, but the copper-resistant genes (*pco*A, *pco*B*, pco*C*, pco*D*, pco*E, and *Cue*O) were unable to detect in all strains (Tables 2, 3). Furthermore, plasmid full sequence analysis showed that the conjugative plasmid from TC4-2 was a circular IncFII plasmid of 71,813 bp with a G + C content of 52% and contained three resistance genes, namely, *bla*_CTX−M−1_, *bla*_CTX−TEM_, and *fos* A (Figure 1).

**Table 2:** Resistant genes in donor and recipient *E. coli* strains, and the corresponding transconjugant.

**Strains**	**Resistance genes**							
	*bla*_CTX−M−1_	*bla*_CTX−M−9_	***pco*** **A**	***pco*** **B**	***pco*** **C**	***pco*** **D**	***pco*** **E**	***Cue*** **O**
EC600	–	–	–	–	–	–	–	–
4	+	+	–	–	–	–	–	–
TC4-2	+	–	–	–	–	–	–	–

**Table 3:** Antibiotic and copper susceptibility of donor and recipient *E. coli* strains, and the corresponding transconjugant.

**Strains**	**Antibiotics (**μ**g/mL)**								**Cu (mmol/L)**
	**CTX**	**CTO**	**NAL**	**CIP**	**AMP**	**GEN**	**TET**	**FLO**	
EC600	0.063	0.5	>128	0.125	8	0.5	1	4	20
4	>16	32	4	0.008	>64	0.5	2	4	20
TC4-2	>16	16	>128	0.125	>64	0.5	1	4	24

CTX, cefotaxime; CTO, ceftiofur; NA, nalidixic acid; CIP, ciprofloxacin; AMP, ampicillin; GEN, gentamicin; TET, tetracycline; FLO, florfenicol.

### High copper improved the conjugation frequency *in vitro*

**Figure 2:**
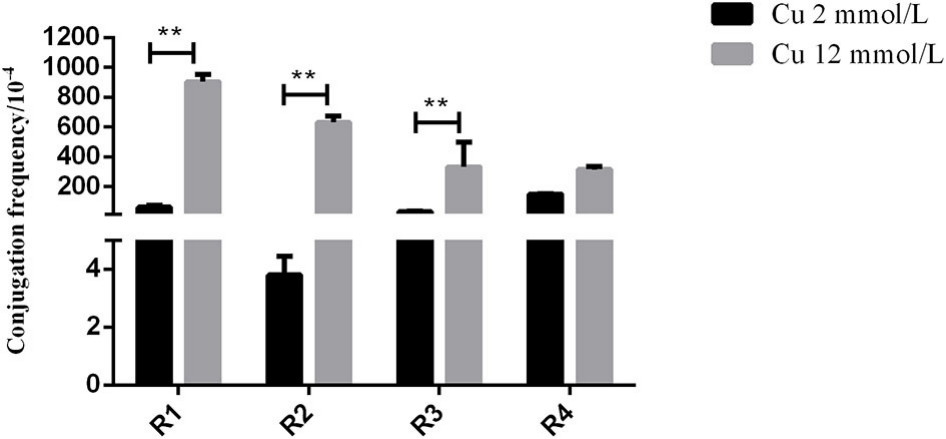
The effects of different copper levels on conjugation frequency. Error bars represent standard error of the means. **means *P* < 0.01.

In the *in vitro* test, the inoculation strain (the CTO-resistant TC4-2 strain harboring a conjugative plasmid carrying a gene conferring resistance *bla*_CTX−M−1_) was used as the donor, and the CTO-susceptible *E. coli* strains isolated from rats were used as the recipient. The results showed that the transfer rates of the conjugation induced by the H^−^ (12 mM) group were significantly higher than that of the low copper (2 mM) group (R1, R2, and R3) (*p* < 0.01) (Figure 2).

### High copper had a positive effect on the maintenance of cefotaxime-resistant *E. coli*

**Figure 3:**
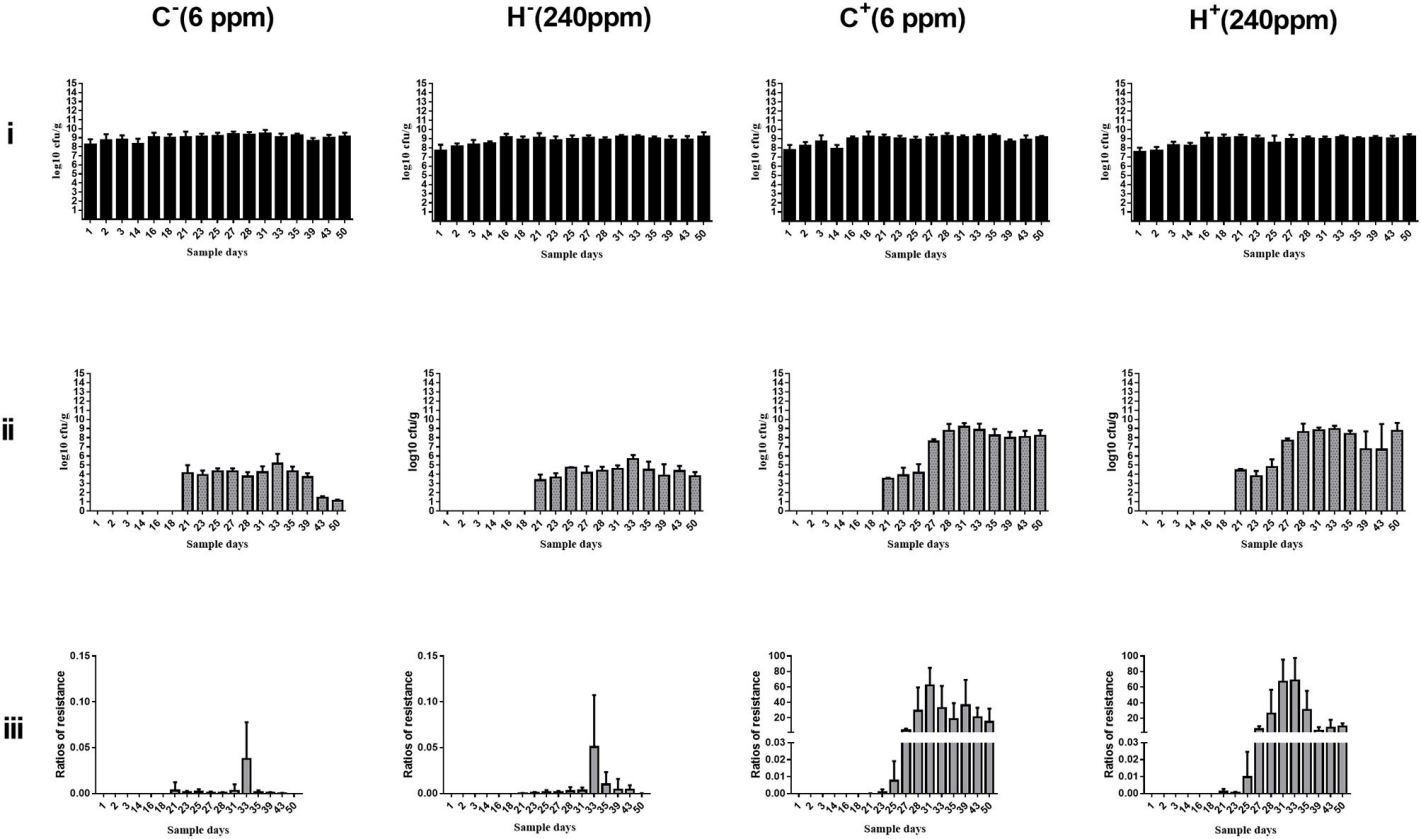
Changes in culturable/cefotaxime-resistant *E. coli* numbers in the individual fecal samples in different groups. **(i)** Change in culturable *E. coli* numbers of the fecal samples in different groups. **(ii)** Change in cefotaxime-resistant *E. coli* numbers of the fecal samples in different groups. **(iii)** Change in ratios of cefotaxime-resistant isolates of the fecal samples in different groups. *E. coli* TC4-2 was inoculated on D20, D22, D24, D26, D27, and D28 in all groups.

The change in cefotaxime-susceptible/cefotaxime-resistant *E. coli* numbers in the individual fecal samples in the different treatment groups is shown in Figure 3. There were no significant differences among all treatment groups before transconjugants and CTO treatment. No cefotaxime-resistant *E. coli* was detected in fecal samples before TC4-2 inoculation. However, after inoculation of TC4-2 (D21), all groups were found cefotaxime-resistant *E. coli* isolates. The ratios of resistant bacteria began to decrease from D31 to D50 in C^−^ and H^−^ groups. However, compared with the C^−^ group, the rate of ratios in the H^−^ group decreased. The mean cefotaxime-resistant *E. coli* numbers in the fecal samples are given in Table 4. The statistical analysis also showed a trend that the rate of disappearance of the *bla*_CTX−M−1_ gene was slower in the H^−^ group compared with the C^−^ group from D31 to D50 (Table 4). As for C^+^ and H^+^, the number of the cefotaxime-susceptible *E. coli* population had a transient decrease on D28 in C^+^ and H^+^ groups, but was soon restored after cessation of dosing. The H^+^ group, restoring the original level for 3 days, was faster than the C^+^ group, which took 4 days (Figure 3). After administration of CTO, the counts of CTX-resistant *E. coli* in the C^+^ group were significantly higher than that in the corresponding control group (C^+^ vs. C^−^; H^+^ vs. H^−^) (*p* < 0.01) (Table 4).

**Table 4:** Mean cefotaxime-resistant isolates numbers in the individual fecal samples (log10 CFU/g).

**Group**	**Sample days**										
	**D21**	**D23**	**D25**	**D27**	**D28**	**D31**	**D33**	**D35**	**D39**	**D43**	**D50**
C– (6 ppm)	1.02	2.93	2.17	2.72^b^	3.29^b^	3.69^b^	5.15^b^	4.32^b^	1.85^b^	1.35 ^b^	1.11 ^b^
H– (240 ppm)	1.26	1.82	1.18	2.61^b^	2.20^b^	4.02^b^	5.66^b^	4.51^b^	0.97^b^	3.27^b^	1.42^b^
C^+^ (6 ppm)	0.45	1.46	2.61	7.58^a^	8.73^a^	9.17^a^	8.84^a^	8.24^a^	7.98^a^	8.09^a^	8.20^a^
H^+^ (240 ppm)	1.66	1.89	2.39	7.65^a^	8.63^a^	8.83^a^	8.96^a^	8.42^a^	6.76^a^	6.71^a^	8.74^a^
SEM	0.32	0.35	0.41	0.52	0.59	0.50	0.34	0.37	0.61	0.59	0.71
*P*-value	0.62	0.41	0.60	≤0.01							

Values in a row with different superscripts are significantly different, P < 0.01.

Ceftiofur was given on D26, D27, and D28 to C^+^ and H^+^ groups, and E. coli TC4-2 was inoculated on D20, D22, D24, D26, D27, and D28 in all groups.

### High copper improved the detection ratio of *bla*_CTX-M-1_ gene in putative new transconjugants

The inoculated strain (TC4-2) showed white colonies on MC agar, similar to EC600. However, the putative new transconjugants of conjugation *in vivo* showed red colonies. Both of them were resistant to cefotaxime. In terms of the differences, the two strains could be directly distinguished on MC agar containing cefotaxime. On each sample day, four putative new transconjugants for each rat were selected to detect the *bla*_CTX−M−1_ gene. The results for *bla*_CTX−M−1_ are given in Table 5. All samples collected before inoculation were negative. In the first 3 days after inoculation, most isolates showed white colonies on MC agar, similar to *E. coli* inoculated at first (Figure 3). At D33 and D35, the detection ratio of rats shedding the *bla*_CTX−M−1_ gene in the H^−^ group was significantly higher than thar in the C^−^ group (*P* < 0.05). The total number of samples detected positive from D21 to D57 according to PCR was 74 out of 206 for the C^−^ group vs. 109/169 for the H^−^ group, which indicates that the total detection ratio of the *bla*_CTX−M−1_ gene from D27 to D57 in the H^−^ group (64.50%) was significantly higher than that in the C^−^ group (35.92%) (*P* < 0.001). Moreover, after 3 days of CTO treatment, the rate in the C^−^ group decreased faster than the H^−^ group. However, there was no significant difference between C^+^ and H^+^, except on D31 and D33.

**Table 5:** Numbers and ratios of rats shedding the *bla*_CTX−M−1_ gene, as determined by PCR.

**Group**	**Day**								**Total**
	**D21** + **D23** + **D25**	**D27**	**D28**	**D31**	**D33**	**D35**	**D43**	**D57**	
C^−^ (6 ppm)	4/13 (30.77%)	6/7^a^ (85.71%)	3/24^b^ (12.50%)	22/27^a^ (81.48%)	16/32^c^ (50.00%)	11/21^b^ (52.38%)	8/12^ab^ (66.67%)	3/16^b^ (18.75%)	74/206^c^ (35.92%)
H^−^ (240 ppm)	2/5 (40.00%)	2/2^a^ (100.00%)	6/27^b^ (22.22%)	23/25^a^ (92.00%)	28/32^ab^ (87.50%)	24/29^a^ (82.76%)	16/22^ab^ (72.73%)	6/24^b^ (25.00%)	109/169^b^ (64.50%)
C^+^ (6 ppm; +)	3/8 (37.50%)	18/18^a^ (100.00%)	24/30^a^ (80.00%)	23/32^a^ (71.88%)	20/28^bc^ (71.43%)	15/16^a^ (93.75%)	29/32^a^ (90.63%)	28/32^a^ (87.50%)	157/193^a^ (81.35%)
H^+^ (240 ppm; +)	0/5 (0.00%)	21/22^a^ (95.45%)	19/29^a^ (65.52%)	18/32^b^ (56.25%)	30/32^a^ (93.75%)	25/31^a^ (80.65%)	18/32^b^ (56.25%)	24/24^a^ (100.00%)	156/207^a^ (75.36%)

Values in a row with different superscripts are significantly different (P < 0.05).

Escherichia coli TC4-2 was inoculated on D20, D22, D24, D26, D27, and D28 in all groups.

### TC4-2 *E. coli* isolates transferred resistant genes to indigenous sensitive isolates by conjugation

Based on the results of animal design, the cefotaxime-sensitive and -resistant strain isolates from rat's fecal samples of the C^−^ and H^−^ groups were analyzed for homology. Similarities in the patterns of SmaI fragments were observed between the new transconjugants and indigenous strains, and some strains appeared to be closely related to the dendrogram constructed from the BioNumerics.

**Figure 4:**
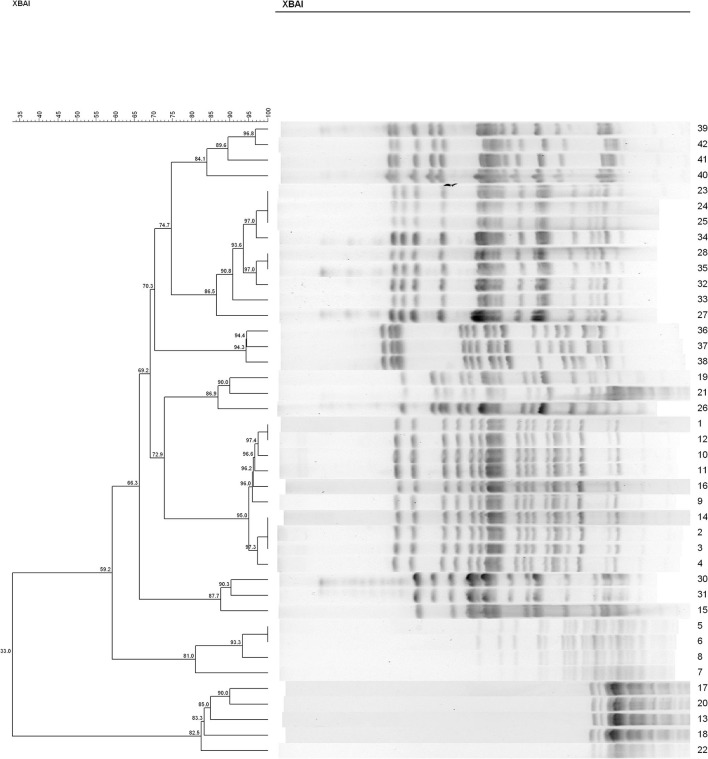
The PFGE of cefotaxime-sensitive or -resistant isolates from one rat's fecal samples in C^−^ group. Numbers 1–34 indicates indigenous sensitive *E. coli* isolates; numbers 35–42 indicate *bla*_CTX−M_-positive *E. coli* isolates.

**Figure 5:**
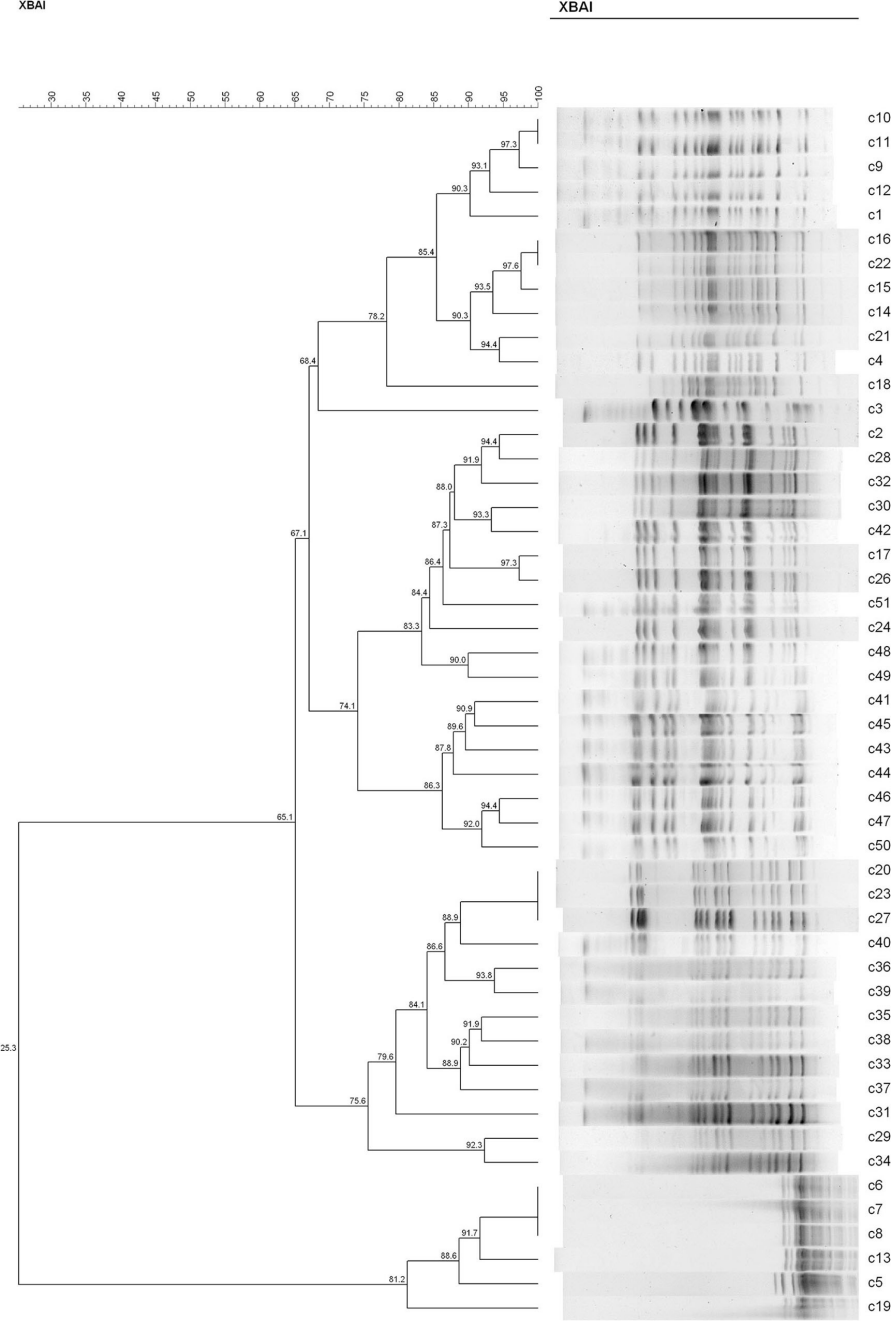
The PFGE of cefotaxime-sensitive or resistant isolates from one rat's fecal samples in H^−^ group. Numbers c1–c31 indicate indigenous sensitive *E. coli* isolates; numbers c32–c51 indicate *bla*_CTX−M_-positive *E. coli* isolates.

In both groups, we found recipient strains (the indigenous sensitive *E. coli*) that were homologous to the transconjugants (the *bla*_CTX−M_-positive *E. coli*). The results showed that nine different PFGE types were detected among the 34 sensitive *E. coli* isolated from rat's fecal samples in the C^−^ group (similarity is < 85%) (Figure 4). In this group, No. 28 and No. 35 had a similarity coefficient of 100% and the PFGE pattern was almost identical (No. 35 was more than one band at about 67 kb position than No. 28). Similarly, 9 different PFGE types were detected among the 31 sensitive *E. coli* isolated from rat's fecal samples in the H^−^ group (c30 and c42, 93.3%; c29 and c34, 92.3%; c2/c2 and c32, 91.9%; c20/c23/c27 and c40, 88.9%) (Figure 5).

**Figure 6:**
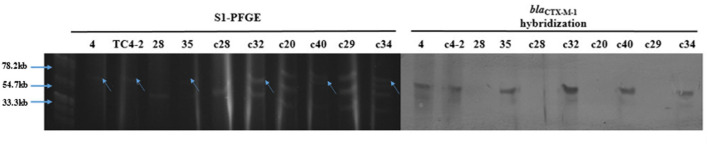
S1-PFGE and Southern-blot hybridization of the donor and recipient isolates, and the corresponding transconjugants.

The results of S1-PFGE showed that the *bla*_CTX−M−1_ gene was present on the plasmid of about 67 kb (indicated by the arrows in Figure 6). Southern-blot hybridization showed that the hybridization strip appeared in the donor strain for inoculum strain (strain No. 4), TC4-2, and *bla*_CTX−M_-positive *E. coli* but not in the corresponding recipient strains. In the C^−^ group, strain No. 35 was found to have one more hybridization strip located at 67 kb than strain No. 28 but less than one strip at 43 kb. It could be presumed that there might be a loss of plasmid during the transfer. The c32, c40, and c34 isolated from the H^−^ group also had an extra band at the 67 kb position compared to the corresponding recipients, i.e., c20, c28, and c29. Besides, after conjugation, the transconjugant c40 lost a plasmid with a size of 55 kb compared with its recipient c20. Southern hybridization also confirmed that the *bla*_CTX−M−1_ gene was located in plasmids with sizes of about 67 kb, which could be transferred to the transconjugants by conjugation and expressed effectively in the recipient strains.

### The new transconjugants showed the similar antibiotic and copper susceptibilities to TC4-2 *E. coli* and the indigenous sensitive isolates

The overall MIC distribution of TC4-2 *E. coli* strains, the indigenous sensitive isolates, and the new corresponding transconjugants for the eight antibiotics and copper are illustrated in Table 6. The MIC of antibiotics varied according to the success of conjugation. The MICs of the new transconjugants to cefotaxime and CTO were higher than those of the corresponding indigenous sensitive isolates (28 and 35, c28 and c32, c20 and c40, c29 and c34) but had no significant differences compared with TC4-2 *E. coli* strains. The transconjugants exhibited the same resistance to nalidixic acid as the indigenous sensitive isolates but increasing resistance compared with TC4-2 *E. coli* strains. Besides, the resistance to ampicillin of transconjugants was higher than the donor strains. The transconjugants showed similar copper tolerance to the corresponding recipient isolates. However, there is no definite association for copper tolerance among transconjugants.

**Table 6:** Antibiotic and copper susceptibility of donor and recipient isolates, and the corresponding transconjugants.

**Strains**	**Antibiotics (**μ**g/mL)**								**Cu (mmol/L)**
	**CTX**	**CTO**	**NAL**	**CIP**	**AMP**	**GEN**	**TET**	**FLO**	
TC4-2	>16	16	>128	0.125	>64	0.5	1	4	24
28	0.5	1	4	0.007813	4	0.5	2	8	24
35	>32	>32	4	0.007813	>64	0.5	2	8	24
c28	0.5	1	4	0.007813	4	0.25	2	8	24
c32	>32	>32	4	0.007813	>64	0.25	2	8	24
c20	0.25	1	4	0.007813	4	0.5	2	8	20
c40	>32	>32	4	0.007813	>64	0.5	2	8	20
c29	0.5	1	4	0.007813	4	0.5	2	8	20
c34	>32	>32	4	0.007813	>64	0.5	2	8	20

Numbers 28, c20, c28, and c29 indicate indigenous sensitive isolates; numbers 35, c32, c34, and c40 indicate bla_CTX−M_-positive isolates.

CTX, Cefotaxime; CTO, Ceftiofur; NA, Nalidixic acid; CIP, Ciprofloxacin; AMP, Ampicillin; GEN, Gentamicin; TET, Tetracycline; FLO, Florfenicol.

## Discussion

In this study, SPF rats were orally inoculated with a rifampicin-resistant *E. coli* EC600 strain (TC4-2) harboring a conjugative plasmid carrying a gene conferring resistance *bla*_CTX−M−1_. The inoculation of *E. coli* was obtained by conjugating the recipient strain EC600 to 10 isolates harboring *bla*_CTX−M_ from our SD rat herd. It should be noted that all the transconjugants contained the *bla*_CTX−M−1_ gene, but none of them detected *bla*_CTX−M−9_, suggesting that the *bla*_CTX−M−1_-type genes were located more commonly on conjugative plasmids. Such differences have already been described (28). Compared the MICs of the eight antibiotics and copper with the donor strain, the transconjugants showed a significant increase in the resistance levels of cephalosporin antibiotics (cefotaxime and CTO), indicating that the resistant phenotype was also transferred at the same time as the resistance gene was obtained. Moreover, the transconjugants exhibited the same resistance to ampicillin with the donor strain; therefore, the resistance to ampicillin was also co-transferred. Juelsgaard et al. (29) also found that strains which were resistant to cephalosporin always showed an ampicillin-resistant profile. We also found that the pTC4-2 contained three resistance genes, namely, *bla*_CTX−M−1_, *bla*_CTX−TEM_, and *fos* A. Hou et al. (30) reported that plasmid-mediated fosfomycin resistance has been frequently detected among CTX-M-producing isolates of animals in China. The authors implied that the conjugative plasmid played a very important role in the propagation of bacterial resistance genes, and the fact that multiple drug resistance genes may be located on the same plasmid exacerbated the increasingly severe tolerance situation from the side. Plasmid pTC4-2 belonged to the IncFII type consistent with those of previous studies, in which *bla*_CTX−M_ genes were found to be located predominantly in Inc FII-type plasmids in *E. coli* isolates recovered from food animals (31).

Fecal samples obtained from rats were used for the enumeration of total *E. coli* on Mac plates and resistant *E. coli* on cefotaxime-Mac plates. Culture results were not significantly different for C^−^ and H^−^ groups before and after inoculation of TC4-2, as well as for C^+^ and H^+^ groups before CTO treatment. After administration of CTO, the average counts of total coliforms had a transient decrease in C^+^ and H^+^ groups, but then soon restored after cessation of the dosing. The H^+^ group restoring the original level was faster than the C^+^ group. There were no cefotaxime-resistant *E. coli* strains isolated from the samples before inoculation. Besides, after CTO treatment, the counts of cefotaxime-resistant *E. coli* in the CTO group (C^+^ and H^+^) were significantly higher than that in the corresponding control group (C^−^ and H^−^) (*p* < 0.01), suggesting that the antibiotics accelerated the dissemination of conjugative plasmid carrying a gene conferring resistance *bla*_CTX−M−1_ *via* conjugation. Under the pressure of antibiotics, the intestinal tract became a great platform for the spread of antibiotic-resistant genes, where the *bla*_CTX−M−1_-producing *E. coli* of inoculated origin (TC4-2) transferred the resistant gene to indigenous sensitive isolates *via* conjugation. Although the number of *E. coli* can gradually return to normal levels when the antibiotic pressure was removed, the resistant gene would persist in harboring the intestinal indigenous bacteria in the intestinal tract for a long time. In the absence of antibiotic selective pressure in the environment, the cefotaxime-resistant *E. coli* in C^−^ and H^−^ groups decreased and gradually disappeared, attributing to the loss of a plasmid-carrying resistant gene in the transmission process. The results of cefotaxime-Mac *E. coli* count showed that the maintenance ability of the H^−^ group was higher than that of the C^−^ group, but there was no difference in the H^+^ group compared with the C^+^ group, indicating that only high copper had a certain degree of effect on the maintenance of resistant genes, but in the combination of high copper and CTO, the effects of antibiotics on maintenance were more obvious.

According to PCR, the detection rate of the *bla*_CTX−M−1_ gene in C^+^ and H^+^ groups was maintained at a higher level compared with C^−^ and H^−^ group during the entire trial period (*P* < 0.05). Fleury et al. (32) showed that the level of *bla*_CTX−M−1_, after administration of CTO hydrochloride (5 mg/kg) by intramuscular injection for SPF piglets harboring *E. coli* carrying *bla*_CTX−M−1_, was significantly higher than the non-treated group (*P* < 0.01). The total detection ratio of the H^−^ group was higher than that of the C^−^ group. The disappearance rate of the *bla*_CTX−M−1_ gene in the H^−^ group was slower than that of the C^−^ group after administration of CTO, while C^+^ and H ^+^ groups did not show a similar difference. The result was consistent with CTX-Mac *E. coli* count. The copper levels of feed and samples of fecal collected from rats were detected (Supplementary Additional file 1). The results showed that with the prolonging feeding time of high-copper diets, the level of copper in the feces increased and prolonged at a high level, indicating that the animal model at different levels of copper was constructed successfully.

To verify the effect of high copper on the horizontal transfer of the *bla*_CTX−M−1_ gene, the conjugation assay *in vitro* was carried out to study the changes in resistant plasmid conjugation frequency under different copper levels. The results showed that the transfer rate of *bla*_CTX−M−1_-positive donor (TC4-2) to receptor (R1, R2, R3, and R4) in the H^−^ group was significantly higher than that of the low copper group (*P* < 0.01), which further confirmed that high copper increased the conjugation rate of resistant plasmid *in vitro* and had a certain effect on accelerating the spread of resistance genes.

Only high copper had a certain degree of effect on the maintenance of resistant genes, but in the combination of high copper and CTO, the effects of antibiotics on maintenance were more obvious. To further study the effect of copper on drug-resistant genes, the cefotaxime-sensitive and *bla*_CTX−M−1_-positive *E. coli* isolates from rat's fecal samples of the C^−^ and H^−^ groups were analyzed the homology by PFGE. Talon et al. (33) explained that strains with a similarity coefficient of more than 85% were considered the same source when PFGE analysis was performed and that more than 50% of the bands were not associated with epidemiology. Tenover et al. (27) documented that an isolate was considered to be possibly related to the outbreak strain if its PFGE pattern differs from the outbreak pattern by changes consistent with two independent genetic events. The results of XbaI-PFGE showed that 34 *E. coli* isolates belonged to nine cluster analysis profiles (< 85% similarity), which were divided into two distinct clones (< 50% similarity). The results also showed that 9 different PFGE types were detected among the 31 sensitive *E. coli* isolated from rat's fecal samples in the H^−^ group. The PFGE analysis of the two groups' isolates in the rat intestinal tract was very homogeneous, revealing high genetic homology. Besides, the addition of high copper levels did not make a certain PFGE type achieve a dominant position in the rat intestine.

The indigenous sensitive isolates (the recipient strain) homologous to the corresponding *bla*_CTX−M_-positive isolates of rat feces (the donor strain) were found by PFGE in C^−^ and H^−^ groups, respectively. Compared with the C^−^ group, the H^−^ group had more resistant isolates which had found the corresponding homologous susceptible strains in the same group. Based on the results of PFGE, the MICs of eight antibiotics were compared among the TC4-2 *E. coli* isolates (the recipient strain *in vivo*), the indigenous sensitive isolates (the donor strain *in vivo*), and the *bla*_CTX−M_-positive isolates that were red in MC agar (the transconjugant *in vivo*). The results showed that the MICs of the transconjugants to cefotaxime and CTO were higher than those of the indigenous sensitive isolates, suggesting that the TC4-2 *E. coli* isolates transferred resistant genes and phenotypes to indigenous sensitive isolates.

The transconjugants and the corresponding recipient strains, as well as the inoculation strain TC4-2 and the donor strain No. 4 (the donor strain of the inoculation strain), were subjected to S1-PFGE and Southern-blot hybridization. The results showed that TC4-2 and strain No. 4 showed a single band at about 67 kb after S1 nuclease digestion, that is, the *bla*_CTX−M−1_ gene was located on this plasmid. After digestion of XbaI-PFGE typing resistant transconjugants and their homologous susceptibility strains, most of the rat fecal sensitive isolates carried a variety of different sizes of plasmids, and the donor strains could transfer conjugative plasmids carrying the *bla*_CTX−M−1_ gene to the receptor, but there was a loss of the recipient plasmid during the conjugation. The hybridization bands were obtained by Southern blotting with the *bla*_CTX−M−1_ gene gel recovered product labeled with digoxigenin, and the clones 35, c32, c40, and c34 were obtained on strain No. 4, TC4-2, and the animal test. Using digoxin-labeled *bla*_CTX−M−1_ gene gel product as a probe for Southern hybridization, the hybridization bands, showing single copy, were obtained from strain No. 4, TC4-2, and some new transconjugants *in vivo* (35, c32, c40, and c34), while no bands were present on the recipient strains 28, c28, c20, and c29, further confirming that the *bla*_CTX−M−1_ gene was derived from the inoculated strain TC4-2.

It has been reported that the addition of high copper in the feed may play an important role in maintaining and promoting bacterial resistance. Berg et al. (34) found that bacteria with copper resistance and antibiotic resistance (ampicillin and sulfa) were more common in high copper (116.7 mg/kg) soil, and the tolerance to chloramphenicol in Gram-negative bacteria of high copper soil was significantly higher than that of low copper (8.7 mg/kg) soil. Baker et al. (3) believe that the environment of heavy metals has a synergistic impact on the formation of antibiotic resistance, which was called co-resistance, referring to the heavy metal resistance gene and drug resistance gene located on the same mobile genetic elements (plasmids, transposons, and so on) (35) such as the streptomycin resistance gene and the mercury resistance gene located on the same plasmid (36). In our study, *bla*_CTX−M−1_-positive strains (TC4-2) had a higher tolerance to copper (24 mM), but there were no plasmid-mediated copper-resistant genes (*pco* and *Cue*O) detected, showing that the *bla*_CTX−M−1_ gene was not located on the same plasmid as the copper-resistant gene. In addition, the *bla*_CTX−M_-positive isolates of rat feces showed a similar copper tolerance to the corresponding recipient isolates but did not show a definite association for copper tolerance among themselves. It indicated that the copper resistance gene and drug resistance gene did not locate on the same mobile genetic elements and that *E. coli* may transfer copper resistance and cephalosporin resistance through other mechanisms.

The co-selection mechanisms of antibiotic and copper resistance included not only co-resistance but also cross-resistance, co-regulation, and biofilm induction (3). For many of these mechanisms, the role of copper has already been reported; for instance, when the heavy metal tolerance mechanism was applied to bacteria, a membrane transport system is usually described as non-specific in terms of its substrates, which can also engender antibiotic resistance or vice versa (13). Besides, Harrison et al. (37) reported that, under the conditions of oxidative stress caused by Cu^2+^, the *soxS* gene whose protein in *E. coli* is a regulatory protein in the AcrAB efflux system is upregulated, which increases the production of AcrAB efflux pump and also increases the antibiotic resistance (chloramphenicol, tetracycline, and novobiocin) of bacteria. However, in our study, the mechanism may be related to bacterial biofilms, which also conduce to the co-selection of antibiotic and heavy metal resistance. A recent report suggests that biofilms can affect the life of cells when the entire population of biofilm-containing microorganisms is exposed to metal ions (35). Other researchers also found that both metal and antibiotic sequestration in the biofilm matrix may be important factors in antibiotic and heavy metal resistance (38). In both *in vitro* and *in vivo* experiments, the results showed that copper could improve the conjugation rates. Research on copper in this area is scarce. However, Ou et al. (39, 40) found similar results on zinc. When mixed with male cells, the incubation in 10^−3^ M Zn^2+^ could increase the ability of F^−^ cells to form mating pairs by increasing the receptor site that resides on the surface of the cell wall so as to improve the conjugation rates. It can explain our results to some extent, but the specific mechanism still needs to be studied further.

## Conclusion

Based on the cultural and molecular methods used on fecal samples, the results of this study might provide valuable information corroborating that high copper facilitated the horizontal transfer and maintenance of the resistant genes, but in the presence of high copper and antibiotics, the effects of antibiotics on maintenance were more obvious. In addition, it was verified that bacteria such as *E. coli* can transfer conjugative plasmids carrying a gene conferring resistance *bla*_CTX−M−1_ to the recipient through conjugation so as to spread the resistant genes among different bacteria. However, the *bla*_CTX−M−1_ gene was not located on the same plasmid as the copper-resistant gene, indicating that *E. coli* may transfer copper resistance and cephalosporin resistance through other mechanisms. The possible association between them poses a serious challenge for antibiotic treatment. More studies are needed to explore the dissemination mechanism of copper resistance and cephalosporin resistance.

## Data availability statement

The datasets presented in this study can be found in online repositories. The names of the repository/repositories and accession number(s) can be found below: https://www.ncbi.nlm.nih.gov/genbank/, OQ459707.

## Ethics statement

The animal study was reviewed and approved by the Committee of Animal Research Institute [Certification No. SYXK (Su) 2011-0036], Nanjing Agricultural University, China.

## Author contributions

WY, MW, RG, FZ, LL, and KL designed the study. MW, RG, and LL conducted the experiment. MW, LL, JH, and RG performed and collected the data. LL and KL analyzed the data and wrote the manuscript. All authors read and approved the final manuscript.
