# Supplementary material for: Effect of high-copper diet on transference of blaCTX−M genes among Escherichia coli strains in rats' intestine
Source: Front Vet Sci. 2023 Mar 9;10:1127816. doi: 10.3389/fvets.2023.1127816 (PMC10033962; doi:10.3389/fvets.2023.1127816)
Supplement: Supplementary file 1 [file Table_1.DOCX]

Supplementary Material

Effect of dietary high copper on transference of blaCTX-M genes among Escherichia coil strains in rats’ intestine

Kang Liu, Linqian Li, Mengwei Weng, Feng Zhang, Rong Guo, Jinhu Huang, Wen Yao*

*** Correspondence:** **Wen Yao**: [yaowen67jp@njau.edu.cn](mailto:yaowen67jp@njau.edu.cn)

Supplementary table: Changes of copper levels in rat fecal samples (mg/kg).

| Group | Sample days | | | SEM | *P*-value |
| --- | --- | --- | --- | --- | --- |
|  | D1 | D27 | D57 |  |  |
| C^-^（6 mg/kg） | 125.87 | 113.94^a^ | 110.89^a^ | 7.93 | 0.73 |
| C^+^（6 mg/kg） | 104.74 | 136.88^a^ | 112.99^a^ | 16.28 | 0.72 |
| H^-^（240 mg/kg） | 91.27^X^ | 4109.58^bY^ | 4215.76^bY^ | 421.74 | ＜0.01 |
| H^+^（240 mg/kg） | 61.44^X^ | 3968.78^bY^ | 3855.33^bY^ | 388.22 | 0.01 |
| SEM | 13.06 | 373.84 | 364.62 |  |  |
| *P*-value | 0.08 | ＜0.01 | ＜0.01 |  |  |

Values in a column with different lowercase superscripts are significantly different (*P*＜0.05), in a row with different capital superscripts are significantly different（*P*＜0.05）.
